# Phenotypic and genotypic characterization of antibiotic resistance of methicillin-resistant *Staphylococcus aureus* isolated from hospital food

**DOI:** 10.1186/s13756-017-0257-1

**Published:** 2017-10-04

**Authors:** Farhad Safarpoor Dehkordi, Hasan Gandomi, Afshin Akhondzadeh Basti, Ali Misaghi, Ebrahim Rahimi

**Affiliations:** 1 Department of Food Hygiene and Quality Control, Faculty of Veterinary Medicine, University of Tehran, Tehran, Iran; 2 Department of Food Hygiene and Public Health, Faculty of Veterinary Medicine, Shahrekord Branch, Islamic Azad University, Shahrekord, Iran

**Keywords:** Methicillin-resistant *Staphylococcus aureus*, Biotypes, Antimicrobial resistance, Hospital food

## Abstract

**Background:**

Pathogenic biotypes of the Methicillin-resistant *Staphylococcus aureus* (MRSA) strains are considered to be one of the major cause of food-borne diseases in hospitals. The present investigation was done to study the pattern of antibiotic resistance and prevalence of antibiotic resistance genes of different biotypes of the MRSA strains isolated from various types of hospital food samples.

**Methods:**

Four-hundred and eighty-five raw and cooked hospital food samples were cultured and MRSA strains were identified using the oxacillin and cefoxitin disk diffusion tests and *mecA*-based PCR amplification. Isolated strains were subjected to biotyping and their antibiotic resistance patterns were analyzed using the disk diffusion and PCR methods.

**Results:**

Prevalence of *S. aureus* and MRSA were 9.69 and 7.62%, respectively. Meat and chicken barbecues had the highest prevalence of MRSA. Prevalence of bovine, ovine, poultry and human-based biotypes in the MRSA strains were 8.10, 8.10, 32.43 and 48.64%, respectively. All of the MRSA strains recovered from soup, salad and rice samples were related to human-based biotypes. MRSA strains harbored the highest prevalence of resistance against penicillin (100%), ceftaroline (100%), tetracycline (100%), erythromycin (89.18%) and trimethoprim-sulfamethoxazole (83.78%). *TetK* (72.97%), *ermA* (72.97%), *msrA* (64.86%) and *aacA-D* (62.16%) were the most commonly detected antibiotic resistance genes.

**Conclusions:**

Pattern of antibiotic resistance and also distribution of antibiotic resistance genes were related to the biotype of MRSA strains. Presence of multi-drug resistance and also simultaneous presence of several antibiotic resistance genes in some MRSA isolates showed an important public health issue Further researches are required to found additional epidemiological aspects of the MRSA strains in hospital food samples.

## Background

Consumption of contaminated food is one of the most common cause of outbreak of food-borne diseases in hospitals [1]. Based on the general weakness of the hospitalized patients and also the possibility of occurrence of suppression in their immune system, hospital foodstuffs should have a high quality and safety [1].

The most cases of food-borne outbreaks in hospitals are occurred due to the consumption of food contaminated with *Staphylococcus aureus* (*S. aureus*) [2, 3]. *Staphylococcus aureus* is commonly found in nose and respiratory system and on the skin [2, 3]. It is responsible for the occurrence of nosocomial and community-acquired infections, food-borne diseases and food poisoning [2, 3]. Occurrence of different types of gastrointestinal diseases which are known by vomiting, nausea, abdominal cramps, weakness and diarrhea and also toxic shock syndrome are attributed to the *S. aureus* strains [2, 3].

One of the most interesting questions about the contamination of foods with *S. aureus* concerns the source of these contamination organisms [4–6]. When foods or some of their ingredients are of animal origin it can be of importance to determine whether the strains of *S. aureus* isolated originate from animals or from humans [4–6]. *S. aureus* can be disseminated in the host’s environment. Presence of several biotypes in the *S. aureus* strains (bovine, ovine, poultry and human) with different microbiological characters has increased the importance of the issue [4–6]. Identification of *S. aureus* biotypes is a practical approach to determine the exact routes of food contamination and also find their microbiological and epidemiological similarities and differences [4–6]. Biotyping of *S. aureus* strains isolated from hospital food samples is essential to trace their origin and their public health significance, to investigate the relationship of the strains, and to determine their diversity within and between samples [4–6].

Food-borne *S. aureus* strains are usually resistant against several types of antibiotics [7–9]. Nowadays, methicillin-resistant *S. aureus* (MRSA) has become a serious problem in hospitals [7–9]. Documented data revealed that about 50-70% of the *S. aureus* strains isolated from the hospital environment were MRSA [7–9]. MRSA strains are responsible for about 100,000 cases of infections with around 20% mortality rate each year in the United States [7]. High pathogenicity of MRSA strains [9], its high resistance to several types of antibiotics [9] and its food-borne aspects [9] have increased the importance of isolation of MRSA in hospital food samples. Staphylococcal food poisoning is an intoxication that results from the consumption of foods containing sufficient amounts of one (or more) preformed enterotoxin [2, 3]. Therefore, the risk of MRSA contaminated food might be due to the important factors like cross-contamination [2, 3, 7].

MRSA strains of both animal and human origins are believed to serve as important reservoirs of antimicrobial resistance genes which can transfer and integrate into the MRSA genome leading to the emergence of new and potentially more resistant strains [10–13]. Documented data revealed that presence of certain antibiotic resistance genes is responsible for occurrence of severe antibiotic resistance [10–13]. Reports showed that high presence of *mecA*, *aacA-D*, *tetK* and *tetM*, *ermA* and *ermC*, *msrA*, *linA* and *vatA*, *vatB* and *vatC* antibiotic resistance genes in the *S. aureus* strains isolated of foodstuffs caused severe occurrence of resistance against methicillin, aminoglycosides, tetracyclines, macrolide–lincosamide-streptogramin B, macrolides, lincosamides and streptogramin A groups of antibiotics, respectively [10–13].

Reports of methicillin-resistant strains are challenging due to the large proportion of methicillin-resistant strains and increasing numbers of isolates reinforcing the need to revise their importance to food safety [10–14]. Therefore, screening of these elements is important for public health and despite the importance of such a screen, limited data are available for MRSA at the species level among the hospital food samples.

MRSA strains have been tested in hospital food samples to assess microbiological safety, sanitation conditions during processing, and storage quality of products. High pathogenicity of MRSA strains and general weakness of hospitalized patients make it necessary to assess the presence of MRSA strains in hospital food samples. The current research was done to study the prevalence rate and antimicrobial resistance properties of the MRSA biotypes isolated from various types of raw and cooked hospital food samples in Iran.

## Methods

### Samples

From June 2015 to June 2016, a total of 485 various types of raw and cooked hospital food samples including raw meat (*n* = 38), raw chicken (*n* = 37), raw fish (*n* = 9), meat barbecue (*n* = 31), chicken barbecue (*n* = 82), grilled fish (*n* = 19), soup (*n* = 94), salad (*n* = 56) and cooked rice (*n* = 119) were randomly collected from the big hospitals of the Isfahan province, Iran. Samples were immediately transferred to the Food Hygiene Research Center of the Islamic Azad University of Shahrekord in cooler with ice-packs. All food samples showed normal physical characters including odor, color and consolidation.

### Isolation and identification of *S. aureus*

Each sample was aseptically weighed in an analytical balance and 25 g were transferred into a sterile plastic bag. Then, 225 mL of buffered peptone water (Merck, Germany) was added and homogenized in a Stomacher Bagmixer 400 W (Interscience, Saint-Nom, France) for 2 min. Five milliliter aliquot of the enriched homogenate was transferred into 50 mL Trypticase Soy Broth (TSB, Merck, Germany) supplemented with 10% NaCl and 1% sodium pyruvate. After incubation at 35 °C for 18 h, a loopful of the culture was plated onto Baird-Parker agar supplemented with egg yolk tellurite emulsion (Merck, Germany) and incubated overnight at 37 °C. Black shiny colonies surrounded by 2 to 5-mm clear zones were further identified on the basis of Gram staining, hemolytic activity on sheep blood agar (Merck, Germany), catalase activity, coagulated test (rabbit plasma), oxidase test, glucose O/F test, resistance to bacitracin (0.04 U), mannitol fermentation on Mannitol salt agar (Merck, Germany), urease activity, nitrate reduction, phosphatase, deoxyribonuclease (DNase, Merck, Germany) test, voges-proskaver (Merck, Germany) test and carbohydrate (xylose, sucrose, trehalose and maltose, fructose, lactose, mannose) fermentation tests [11].

### Identification of Methicillin-resistant *Staphylococcus aureus* strains

Cefoxitin (30 μg) and oxacillin (1 μg) susceptibility tests were used to distinguish the MRSA strains from *S. aureus* isolates of hospital food samples. All tests were performed using the guidelines of the Clinical and Laboratory Standards Institute (CLSI) [15].

MRSA isolates were identified another time using the PCR-based amplification of *mecA* gene. MRSA strains were sub-cultured on TSB media (Merck, Germany) and further incubated for 48 h at 37 °C. Genomic DNA was extracted from bacterial colonies using the DNA extraction kit (Fermentas, Germany) according to manufacturer’s instruction.

The PCR reactions were performed in a total volume of 25 μL, including 1.5 mM MgCl2, 50 mM KCl, 10 mM Tris-HCl (pH 9.0), 0.1% Triton X-100, 200 μM dNTPs each (Fermentas, Germany), 2.5 μL PCR buffer (10×), 2.5 mM of each primer *mecA1* (5′-ACGAGTAGATGCTCAATATAA-3′) and *mecA2* (5′-CTTAGTTCTTTAGCGATTGC-3′) (Gen Bank Accession Number NC_003923M), 1.5 U of Taq DNA polymerase (Fermentas, Germany) and 5 μL of the extracted DNA template of the MRSA isolates. The PCR cycling conditions were as follows: initial denaturation at 94 °C for 3 min, followed by 30 cycles of 94 °C for 30 s, 60 °C for 30 s, and 72 °C for 30 s, followed by an extra cycle of annealing at 60 °C for 30 s, and a final extension at 72 °C for 5 min.

### Biotyping of the MRSA strains

Biotyping of the MRSA strains was done according to the method described by Devriese (1984) [16] with minor modifications. MRSA isolates were examined for production of staphylokinase and β-hemolysin, coagulation of bovine plasma and ability to growth on crystal violet agar. Staphylokinase production of the MRSA isolates were studied by incubating on bovine fibrin plates with or without dog serum as a plasminogen source. Staphylokinase production is determined by observation of lear zones on the bovine fibrin plates with dog serum. MRSA isolates were also cultured on 5% sheep blood agar media for determination of production of Beta-haemolysin. Development of broad discolored zones with sharp edges clearing at 4 °C is a marker for production of Beta-haemolysin. Coagulation of bovine plasma is studied by adding 0.1 mL of an overnight culture of the MRSA isolates in Brain Heart Infusion broth (Merck, Germany) to tubes with diluted (1:3) bovine plasma. Occurrence of big clots within 6 h were considered as positive reaction. MRSA strains were also streaked into the crystal violet agar media and appearance of A (crystal violet spots with a bright or pale yellow color and yellow spots with blue margins), C (blue or violet growth spots with or without an orange tint) and E (white spots or white growth with a blue hue) types were studied.

### Antibiotic susceptibility test of MRSA strains

Patterns of antimicrobial resistance of the MRSA strains were studied using the simple disk diffusion technique. The Mueller–Hinton agar (Merck, Germany) medium was used for this purpose. Susceptibility of MRSA isolates were tested against several types of antibiotic groups including Penicillins (penicillin (10 μg/disk)), Cephems (ceftaroline (30 μg/disk),), Aminoglycosides (gentamicin (10 μg/disk), amikacin (30 μg/disk), kanamycin (30 μg/disk)), Macrolides (azithromycin (15 μg/disk) and erythromycin (15 μg/disk)), Tetracyclines (tetracycline (30 μg/disk), doxycycline (30 μg/disk)), Fluoroquinolones (ciprofloxacin (5 μg/disk) and levofloxacin (5 μg/disk)), Lincosamides (clindamycin (2 μg/disk)), Folate pathway inhibitors (trimethoprim-sulfamethoxazole (25 μg/disk)), Phenicols (chloramphenicol (30 μg/disk)) and Ansamycins (rifampin (5 μg/disk)) antibiotic agents (Oxoid, UK) using the instruction of Clinical and Laboratory Standards Institute [17]. The plates containing the discs were allowed to stand for at least 30 min before incubated at 37 °C for 24 h. The diameter of the zone of inhibition produced by each antibiotic disc was measured and interpreted using the CLSI zone diameter interpretative standards [17]. *Staphylococcus aureus* ATCC 25923 was used as quality control organism in antimicrobial susceptibility determination.

### PCR-based amplification of antibiotic resistance genes

Table 1 represents the list of primers and PCR conditions used for amplification of antibiotic resistance genes in the MRSA strains isolated from various types of hospital food samples [18, 19]. A programmable DNA thermo-cycler (Eppendorf Mastercycler 5330, Eppendorf-Nethel-Hinz GmbH, Hamburg, Germany) was used in all PCR reactions.

**Table 1:** Target genes, oligonucleotide primers and PCR conditions used for detection of antibiotic resistance genes in the MRSA strains isolated from various types of hospital food samples

Target gene	Primer sequence (5′-3′)	PCR product (bp)	PCR programs	PCR volume (50 μL)
*AacA-D*	F: TAATCCAAGAGCAATAAGGGC R: GCCACACTATCATAACCACTA	227	1 cycle: 94 ^0C^ ------------ 5 min. 25 cycle: 94 ^0C^ ------------ 60 s 55 ^0C^ ------------ 70 s 72 ^0C^ ------------ 60 s 1 cycle: 72 ^0C^ ------------ 10 min	5 μL PCR buffer 10X 1.5 mM Mgcl_2_ 200 μM dNTP (Fermentas) 0.5 μM of each primers F & R 1.25 U Taq DNA polymerase (Fermentas) 2.5 μL DNA template
*ermA*	F: AAGCGGTAAACCCCTCTGA R: TTCGCAAATCCCTTCTCAAC	190		
*ermC*	F: AATCGTCAATTCCTGCATGT R: TAATCGTGGAATACGGGTTTG	299		
*tetK*	F: GTAGCGACAATAGGTAATAGT R: GTAGTGACAATAAACCTCCTA	360		
*vatC*	F: AAAATCGATGGTAAAGGTTGGC R: AGTTCTGCAGTACCGGATTTGC	467		
*tetM*	F: AGTGGAGCGATTACAGAA R: CATATGTCCTGGCGTGTCTA	158	1 cycle: 94 ^0C^ ------------ 6 min. 34 cycle: 95 ^0C^ ------------ 50 s 55 ^0C^ ------------ 70 s 72 ^0C^ ------------ 60 s 1 cycle: 72 ^0C^ ------------ 8 min	5 μL PCR buffer 10X 2 mM Mgcl_2_ 200 μM dNTP (Fermentas) 0.5 μM of each primers F & R 1.5 U Taq DNA polymerase (Fermentas) 5 μL DNA template
*vatA*	F: TGGTCCCGGAACAACATTTAT R: TCCACCGACAATAGAATAGGG	268		
*msrA*	F: GGCACAATAAGAGTGTTTAAAGG R: AAGTTATATCATGAATAGATTGTCCTGTT	940	1 cycle: 94 ^0C^ ------------ 6 min. 34 cycle: 95 ^0C^ ------------ 60 s 50 ^0C^ ------------ 70 s 72 ^0C^ ------------ 70 s 1 cycle: 72 ^0C^ ------------ 8 min	5 μL PCR buffer 10X 2 mM Mgcl_2_ 150 μM dNTP (Fermentas) 0.75 μM of each primers F & R 1.5 U Taq DNA polymerase (Fermentas) 3 μL DNA template
*vatB*	F: GCTGCGAATTCAGTTGTTACA R: CTGACCAATCCCACCATTTTA	136	1 cycle: 94 ^0C^ ------------ 6 min. 35 cycle: 95 ^0C^ ------------ 50 s 55 ^0C^ ------------ 70 s 72 ^0C^ ------------ 80 s 1 cycle: 72 ^0C^ ------------ 10 min	5 μL PCR buffer 10X 2 mM Mgcl_2_ 150 μM dNTP (Fermentas) 0.75 μM of each primers F & R 1.5 U Taq DNA polymerase (Fermentas) 3 μL DNA template
*linA*	F: GGTGGCTGGGGGGTAGATGTATTAACTGG R: GCTTCTTTTGAAATACATGGTATTTTTCGA	323	1 cycle: 94 ^0C^ ------------ 6 min. 30 cycle: 95 ^0C^ ------------ 60 s 57 ^0C^ ------------ 60 s 72 ^0C^ ------------ 60 s 1 cycle: 72 ^0C^ ------------ 10 min	5 μL PCR buffer 10X 2 mM Mgcl_2_ 150 μM dNTP (Fermentas) 0.75 μM of each primers F & R 1.5 U Taq DNA polymerase (Fermentas) 3 μL DNA template

### Statistical analysis

Statistical analysis was done using the SPSS 21.0 statistical software (SPSS Inc., Chicago, IL, USA). Chi-square test and Fisher’s exact two-tailed test were used to assess any significant relationship between prevalence of MRSA strains, their biotypes, antibiotic resistance genes and antibiotic resistance pattern. *P* value <0.05 was considered as statistical significant level.

## Results

In this study, the prevalence of *S. aureus* strains in various types of raw and cooked hospital food samples were investigated and the results are shown in Table 2. Forty-seven out of 485 hospital food samples (9.69%) were positive for *S. aureus*. Furthermore, the prevalence of *S. aureus* in raw food samples with animal origin, cooked food samples with animal origin and cooked food samples without animal origin were 23.80, 9.09 and 4.08%, respectively. Chicken meat samples had the highest prevalence of *S. aureus* (27.02%) among all studied raw food samples. Meat barbecue samples had the highest prevalence of *S. aureus* (26.31%) among all studied cooked food samples with animal origin. Salad samples had the highest prevalence of *S. aureus* (7.41%) among all studied cooked food samples without animal origin. None of the raw and cooked fish samples were *S. aureus* positive. Statistically significant difference was seen in the prevalence of *S. aureus* between different types of food samples (*P* < 0.05). Moreover, a statistically significant difference was found in the prevalence of *S. aureus* between raw and cooked food samples.

**Table 2:** Prevalence of *S. aureus* and MRSA strains isolated from various types of hospital food samples

Types of samples			No samples collected	N (%) of *S. aureus* positive samples	N (%) of MRSA positive samples
Raw foods	With animal origin	Meat	38	10 (26.31)	6 (50)
		Chicken	37	10 (27.02)	8 (80)
		Fish	9	–	–
		Total	84	20 (23.80)	14 (70)
Cooked foods	With animal origin	Meat barbecue	31	5 (16.12)	5 (100)
		Chicken barbecue	82	7 (8.53)	7 (100)
		Grilled fish	19	–	–
		Total	132	12 (9.09)	12 (100)
	Without animal origin	Soup	94	6 (6.38)	6 (100)
		Salad	56	4 (7.14)	4 (100)
		Rice	119	5 (4.20)	1 (20)
		Total	269	15 (5.57)	11 (73.33)
Total			485	47 (9.69%)	37 (78.72)

Thirty–seven out of 47 *S. aureus* isolates (78.72%) were recognized as MRSA. The prevalence of MRSA strains in raw foods with animal origin, cooked foods with animal origin and cooked foods without animal origin were 16.66, 9.09 and 4.08%, respectively. All of the *S. aureus* isolates of meat barbecue, chicken barbecue, soup and salad samples had complete resistance against methicillin. Rice had the lowest prevalence of MRSA strains (20%). Statistically significant difference was seen in the prevalence of MRSA between different types of food samples (*P* < 0.05). Statistically significant difference was also found in the prevalence of *S. aureus* between raw and cooked food samples (*P* < 0.05).

Table 3 represents the prevalence of different biotypes among the MRSA strains isolated from various types of hospital food samples. The prevalence of bovine, ovine, poultry and human-based biotypes in the MRSA strains isolated from various types of hospital food samples were 8.10, 8.10, 32.43 and 48.64%, respectively. The biotypes of the 2.70% of MRSA strains were determined as unknown. All of the MRSA strains recovered from soup, salad and rice samples were related to human-based biotypes. Statistically significant difference was seen in the prevalence of different biotypes between different types of food samples (*P* < 0.05).

**Table 3:** Prevalence of different biotypes among the MRSA strains isolated from various types of hospital food samples

Types of samples (N of MRSA positive samples)	N (%) of positive samples for each biotype				
	Bovine	Ovine	Poultry	Human	Unknown
Meat (6)	1 (16.66)	3 (50)	1 (16.66)	1 (16.66)	–
Chicken (8)	–	–	5 (62.50)	2 (25)	1 (12.50)
Meat barbecue (5)	2 (40)	–	1 (20)	2 (40)	–
Chicken barbecue (7)	–	–	5 (71.42)	2 (28.57)	–
Soup (6)	–	–	–	6 (100)	–
Salad (4)	–	–	–	4 (100)	–
Rice (1)	–	–	–	1 (100)	–
Total (37)	3 (8.10)	3 (8.10)	12 (32.43)	18 (48.64)	1 (2.70)

Antibiotic resistance pattern of the MRSA strains isolated from animal, human and unknown origins is presented in Table 4. We found that MRSA strains harbored a high prevalence of resistance against penicillin (100%), ceftaroline (100%), tetracycline (100%), erythromycin (86.48%), trimethoprim-sulfamethoxazole (83.78%), doxycycline (81.08%) and gentamicin (81.08%) antibiotic agents. MRSA strains showed low prevalence of resistance against chloramphenicol (29.72%), rifampin (35.13%), levofloxacin (43.24%), kanamycin (43.24%), clindamycin (48.64%), and ciprofloxacin (48.64%) antibiotic agents. Statistically significant difference was seen in the prevalence of antibiotic resistance between various studied biotypes (*P* < 0.05).

**Table 4:** Antibiotic resistance pattern of the MRSA strains isolated from animal, human and unknown origins

Origins (N of MRSA strains)		N (%) isolates resistant to each antibiotic														
		Penicillins	Cephems	Aminoglycosides			Macrolides		Tetracyclines		Fluoroquinolones		Lincosamides	Folate inhibitors	Phenicols	Ansamycins
		P10^a^	Cft	Gen	Amk	Kan	Azi	Ert	Tet	Dox	Cip	Lev	Clin	Tr-Sul	C30	Rif
Animal origins	Meat (5)	5 (100)	5 (100)	5 (100)	2 (40)	1 (20)	1 (20)	5 (100)	5 (100)	4 (80)	1 (20)	1 (20)	1 (20)	4 (80)	2 (40)	1 (20)
	Chicken (5)	5 (100)	5 (100)	4 (80)	3 (60)	2 (40)	2 (40)	4 (80)	5 (100)	4 (80)	2 (40)	2 (40)	2 (40)	4 (80)	2 (40)	1 (20)
	Meat barbecue (3)	3 (100)	3 (100)	2 (66.66)	2 (66.66)	1 (33.33)	–	2 (66.66)	3 (100)	2 (66.66)	–	–	2 (66.66)	3 (100)	1 (33.33)	–
	Chicken barbecue (5)	5 (100)	5 (100)	4 (80)	3 (60)	2 (40)	3 (60)	4 (80)	5 (100)	4 (80)	3 (60)	3 (60)	3 (60)	4 (80)	2 (40)	1 (20)
	Total (18)	18 (100)	18 (100)	15 (83.33)	10 (55.55)	6 (33.33)	6 (33.33)	15 (83.33)	18 (100)	14 (77.77)	6 (33.33)	6 (33.33)	8 (44.44)	15 (83.33)	7 (38.88)	3 (16.66)
Human origins	Meat (1)	1 (100)	1 (100)	1 (100)	1 (100)	1 (100)	1 (100)	1 (100)	1 (100)	1 (100)	1 (100)	1 (100)	1 (100)	1 (100)	–	1 (100)
	Chicken (2)	2 (100)	2 (100)	2 (100)	2 (100)	1 (50)	2 (100)	2 (100)	2 (100)	2 (100)	1 (50)	1 (50)	1 (50)	1 (50)	1 (50)	1 (50)
	Meat barbecue (2)	2 (100)	2 (100)	1 (50)	2 (100)	1 (50)	2 (100)	2 (100)	2 (100)	2 (100)	1 (50)	1 (50)	1 (50)	2 (100)	–	1 (50)
	Chicken barbecue (2)	2 (100)	2 (100)	2 (100)	2 (100)	1 (50)	2 (100)	2 (100)	2 (100)	2 (100)	1 (50)	1 (50)	1 (50)	2 (100)	1 (50)	1 (50)
	Soup (6)	6 (100)	6 (100)	5 (83.33)	4 (66.66)	3 (50)	5 (83.33)	5 (83.33)	6 (100)	4 (66.66)	4 (66.66)	3 (50)	3 (50)	5 (83.33)	1 (16.66)	3 (50)
	Salad (4)	4 (100)	4 (100)	3 (75)	3 (75)	2 (50)	3 (75)	3 (75)	4 (100)	3 (75)	3 (75)	2 (50)	2 (50)	3 (75)	1 (25)	2 (50)
	Rice (1)	1 (100)	1 (100)	–	1 (100)	1 (100)	1 (100)	1 (100)	1 (100)	1 (100)	1 (100)	1 (100)	1 (100)	1 (100)	–	1 (100)
	Total (19)	19 (100)	19 (100)	15 (78.94)	16 (84.21)	10 (52.63)	16 (84.21)	17 (89.47)	19 (100)	16 (84.21)	12 (63.15)	10 (52.63)	10 (52.63)	16 (84.21)	4 (21.05)	10 (52.63)
Unknown origin	Chicken (3)	1 (100)	1 (100)	1 (100)	1 (100)	–	–	1 (100)	1 (100)	1 (100)	–	–	–	1 (100)	–	–
Total (37)		37 (100)	37 (100)	30 (81.08)	26 (70.27)	16 (43.24)	22 (59.45)	32 (86.48)	37 (100)	30 (81.08)	18 (48.64)	16 (43.24)	18 (48.64)	31 (83.78)	11 (29.72)	13 (35.13)

^a^ *P10* penicillin (10 μg/disk), *Cft* ceftaroline (30 μg/disk), *Gen* gentamicin (10 μg/disk), *Amk* amikacin (30 μg/disk), *Kan* kanamycin (30 μg/disk), *Azi* azithromycin (15 μg/disk), *Ert* erythromycin (15 μg/disk), *Tet* tetracycline (30 μg/disk), *Do* doxycycline (30 μg/disk), *Cip* ciprofloxacin (5 μg/disk), *Lev* levofloxacin (5 μg/disk), *Clin* clindamycin (2 μg/disk), *Tr-Sul* trimethoprim-sulfamethoxazole (25 μg/disk), *C30* chloramphenicol (30 μg/disk), *Rif* rifampin (5 μg/disk)

Distribution of antibiotic resistance genes in the MRSA strains isolated from animal, human and unknown origins is presented in Table 5. The most frequently detected antibiotic resistance genes among the MRSA strains were *tetK* (72.97%), *ermA* (72.97%), *msrA* (64.86%) and *aacA-D* (62.16%). Prevalence of *vatC*, *vatB*, *ermC* and *tetM* antibiotic resistance genes were 5.40, 18.91, 27.02 and 27.02%, respectively. The most commonly detected antibiotic resistance gene in the MRSA strains of animal, human and unknown origins were *tetK* (77.77%) and *ermA* (77.77%), *msrA* (68.42%) and *tetK* (100%) and *ermA* (100%), respectively. MRSA strains with unknown origin were only positive for *tetK* and *ermA* antibiotic resistance genes. Statistically significant difference was seen in the prevalence of antibiotic resistance genes between different biotypes (*P* < 0.05).

**Table 5:** Distribution of antibiotic resistance genes in the MRSA strains isolated from animal, human and unknown origins

Origins (N of MRSA strains)		N (%) of isolates harbor each gene									
		*aacA-D*	*tetK*	*tetM*	*msrA*	*ermA*	*ermC*	*vatA*	*vatB*	*vatC*	*LinA*
Animal origins	Meat (5)	4 (80)	5 (100)	2 (40)	4 (80)	4 (80)	1 (20)	3 (60)	1 (20)	–	1 (20)
	Chicken (5)	3 (60)	3 (60)	1 (20)	2 (40)	3 (60)	1 (20)	2 (40)	1 (20)	1 (20)	2 (40)
	Meat barbecue (3)	2 (66.66)	3 (100)	1 (33.33)	2 (66.66)	3 (100)	1 (33.33)	2 (66.66)	1 (33.33)	–	2 (66.66)
	Chicken barbecue (5)	2 (40)	3 (60)	2 (40)	3 (60)	4 (80)	1 (20)	2 (40)	1 (20)	–	2 (40)
	Total (18)	11 (61.11)	14 (77.77)	6 (33.33)	11 (61.11)	14 (77.77)	4 (22.22)	9 (50)	4 (22.22)	1 (5.55)	7 (38.88)
Human origins	Meat (1)	1 (100)	1 (100)	–	1 (100)	1 (100)	–	1 (100)	–	–	1 (100)
	Chicken (2)	2 (100)	2 (100)	1 (50)	2 (100)	1 (50)	1 (50)	1 (50)	1 (50)	–	1 (50)
	Meat barbecue (2)	2 (100)	2 (100)	–	2 (100)	2 (100)	1 (50)	1 (50)	–	–	1 (50)
	Chicken barbecue (2)	2 (100)	2 (100)	1 (50)	2 (100)	2 (100)	1 (50)	1 (50)	1 (50)	–	1 (50)
	Soup (6)	2 (33.33)	2 (33.33)	1 (16.66)	3 (50)	3 (50)	2 (33.33)	2 (33.33)	1 (16.66)	1 (16.66)	2 (33.33)
	Salad (4)	2 (50)	2 (50)	1 (25)	2 (50)	2 (50)	1 (25)	1 (25)	–	–	2 (50)
	Rice (1)	1 (100)	1 (100)	–	1 (100)	1 (100)	–	1 (100)	–	–	1 (100)
	Total (19)	12 (63.15)	12 (63.15)	4 (21.05)	13 (68.42)	12 (63.15)	6 (31.57)	8 (42.10)	3 (15.78)	1 (5.26)	9 (47.36)
Unknown origin	Chicken (1)	–	1 (100)	–	–	1 (100)	–	–	–	–	–
Total (37)		23 (62.16)	27 (72.97)	10 (27.02)	24 (64.86)	27 (72.97)	10 (27.02)	17 (45.94)	7 (18.91)	2 (5.40)	16 (43.24)

## Discussion

*S. aureus* is considered as one of the most common causes of nosocomial infections, as well as the cause of most cases of food poisoning in hospitals [4, 20]. Hospital meals are an indispensable portion of patient care. Safe and complete meals can encourage patients to eat well and giving them the nutrients they need to recover from surgery or illness.

Foodstuff contamination with *S. aureus* may occur directly from infected food-producing animals or may result from poor hygiene during production processes, or the retail and storage of food, since humans may also harbor microorganisms. The current research is the first report of the biotyping and molecular characterization of antibiotic resistance in the MRSA strains isolated from various types of raw and cooked hospital food samples. Findings obtained from this research revealed that the prevalence of *S. aureus* in different types of hospital food samples was 9.69%. The prevalence rate of the *S. aureus* in hospital food samples of our research was higher than that of Spain (6.10%) [21] and Iran (6.42%) [22], Portugal (11.10%) [23] and Brazil (50%) [24].

Investigations conducted in the U.S. as well as numerous other countries, including Canada, Taiwan, China, Denmark, South Korea, Austria, France, Belgium, Italy, and The Netherlands, have isolated MRSA mainly from different types of foods [4, 20]. Costa et al. (2015) [25] revealed that 28.10% of hospital food samples harbored MRSA strains. They showed that the prevalence of MRSA strains in beef, chicken, pork and fish samples were 23.30, 23.30, 37.50 and 30%, respectively which was higher than that found in our study.

Biotyping is a simple method used to trace the origin of *S. aureus* strains isolated from food samples. The results of our study showed that all of the MRSA strains isolated from rice, salad and soup samples were derived from humans. Furthermore, 48.64% of MRSA isolates of hospital food samples had human origin. Generally, the results revealed the role of infected humans in the dissemination and also transmission of MRSA strains to hospital food samples. The role of food handlers in transmission of MRSA strains into the food samples has also been reported by Castro et al. (2016) [23], Ferreira et al. (2014) [24], Costa et al. (2015) [25] and Ayçiçek et al. (2004) [26]. A study which was conducted by Kitai et al. (2005) [27] supported the high prevalence of poultry-based biotypes found in our investigation (71.42%). They reported that about 80% of all *S. aureus* isolates of foodstuffs belonged to the poultry-based biotypes, while prevalence of human-based biotypes was 22.10%. Normanno et al. (2007) [28] revealed that the prevalence of human, ovine, not-host-specific, bovine and poultry-based biotypes of the *S. aureus* isolates of Italian food samples were 50.40, 23.20, 17.60, 7.20 and 1.60%, respectively.

*S. aureus* causes food intoxication and doesn’t lead to food infection [2, 3]. Therefore, the risk of MRSA contaminated hospital food might be due to the cross-contamination. High prevalence of MRSA strains in cooked food samples may be due to the cross-contamination of cooked foods through food handlers and kitchen equipment.

Our results showed that the antibiotic resistance pattern and prevalence of the antibiotic resistance genes were highly dependent to the biotypes of the MRSA strains. The human-based biotypes of the MRSA strains harbored higher prevalence of resistance against human-based antibiotics including ceftaroline, amikacin, kanamycin, azithromycin, doxycycline, ciprofloxacin, levofloxacin, clindamycin and rifampin. Furthermore, animal-based biotypes harbored higher prevalence of resistance against animal-based antibiotics or those which are routine in veterinary medicine including penicillin, gentamicin, erythromycin, tetracycline, trimethoprim-sulfamethoxazole and chloramphenicol. Poultry-based biotypes of the MRSA strains had a higher prevalence of resistance against chloramphenicol (*P* < 0.05). It may be due to the higher prescription of chloramphenicol in aviculture. MRSA strains of our study harbored the highest prevalence of resistance against antibiotics of the penicillins, cephems and tetracyclines groups. There were no previously published data about the relations between biotypes and prevalence of antibiotic resistance in the MRSA strains. Similar antibiotic resistance patterns of the MRSA strains isolated from different types of food and clinical samples have also been reported against aminoglycosides [29–33], cephems [29, 31–33], penicillins [29, 31–33], macrolides [29–33], tetracyclines [29, 31, 32], fluoroquinolones [29–33], lincosamides [29–32], folate inhibitors [29–33], phenicols [29, 31, 32] and ansamycins [29, 31, 32] groups of antibiotics. Fowoyo and Ogunbanwo (2017) [34] reported that the *S. aureus* strains isolated from ready to eat foodstuffs exhibited the high prevalence of resistance against ampicillin (86.70%), trimethoprim–sulfamethoxazole (74.90%), amoxicillin–clavulanic acid (52.50%), cefotaxime (3.50%), oxacillin (35.70%), ciprofloxacin (23.90%), erythromycin (15.70%), gentamicin (11.40%) and ofloxacin (7.10%). Rong et al. (2017) [35] reported that the prevalence of antibiotic resistance in the *S. aureus* strains isolated from different types of food samples against ampicillin, penicillin, amoxicillin–clavulanic acid, cefoxitin, ceftazidime, cefepime, kanamycin, streptomycin, amikacin, gentamicin, norfloxacin, ciprofloxacin, erythromycin, tetracycline, clindamycin, chloramphenicol, trimethoprim-sulfamethoxazole, vancomycin and rifampicin were 88.20, 88.20, 73.90, 8.40, 10.90, 8.40, 22.70, 14.30, 1.70, 4.20, 6.70, 5.00, 53.80, 26.90, 12.60, 7.507.50, 0 and 2.50%, respectively. MRSA strains should resist completely against all types of beta-lactams and penicillins [17], but it is surprising that some studies show that the MRSA strains isolated from food and also clinical samples don’t have complete resistance against several types of beta-lactams and also penicillins [36, 37].

Most of the tetracycline-resistant MRSA strains harbored *tetK* and *tetM* genes. Prevalence of *aacA-D* gene was high among gentamicin, amikacin and kanamycin-resistant MRSA strains. Prevalence of *msrA*, *ermA* and *ermC* and *linA* were also significant among the macrolide, erythromycin and clindamycin-resistant MRSA strains. Therefore, the pattern of the antibiotic resistance of the MRSA strains of hospital food samples was confirmed by the PCR amplification of the specific antibiotic resistance genes. MRSA strains of our study had considerable prevalence of resistance against clindamycin (48.64%). The most imperative mechanism involving resistance against clindamycin is modulated by methylase enzyme which often encoded by *ermA* and *ermC* genes [38]. Prevalence of *ermA* and *ermC* antibiotic resistance genes among the MRSA strains of our research were 72.97 and 27.02%, respectively. Majority of our isolates carried two tetracyclines, two erythromycins, one macrolide and several streptogramin resistance determinants reveals a great diffusion of these types of resistance. *TetK*, *ermA*, *msrA* and *aacA-D* which encode resistance against tetracycline, erythromycin, macrolides and aminoglycosides were the most commonly detected antibiotic resistance genes in the MRSA strains isolated of hospital food samples. The literature survey did not indicate any report on the prevalence of *vatA*, *vatB*, *vatC*, *msrA*, *ermA*, *ermC*, *linA*, *aacA-D*, *tetK* and *tetM* genes among the MRSA strains of hospital food samples. Kumar et al. (2010) [39] reported that the most commonly identified antibiotic resistance genes among the *S. aureus* isolates of food samples were *linA* (51.60%), *msrB* (46.10%), *tetK* (34.40%), *tetM* (34.40%), *msrA* (26.60%) and *aacA-D* (26.60%). Karataş et al. (2017) [40] revealed the higher prevalence of *ermA* than *ermc* antibiotic resistance genes among the clindamycin, erythromycin and telithromycin-resistant and also higher prevalence of *tetM* than *tetK* antibiotic resistance genes among the tetracycline-resistant MRSA strains which were similar to our findings. Our results were also similar with those of the previous research which was conducted by Simeoni et al. (2008) [41]. They reported that the prevalence of *tetM*, *tetO*, *tetK*, *ermA*, *ermB*, *ermC*, *aac*, *blaZ* and *mecA* antibiotic resistance genes amongst the *S. aureus* strains isolated from meat samples were 100, 0, 91.66, 16.66, 33.33, 58.33, 0, 100 and 58.33%, respectively. High prevalence of *tetK* and *tetM* antibiotic resistance genes in the MRSA isolates can be clarified by their usual genetic locations. Presence of *tetK* gene on small multicopy plasmids and *tetM* on conjugative transposons contributes to the spread of these determinants [42]. Some of the MRSA strains harbored *ermC* gene. This gene is often located on small multicopy plasmids which are present in many different staphylococcal species [42]. The *ermA* gene is usually carried by transposons which could explain its high prevalence amongst the MRSA strains. Resistance to aminoglycosides (43.24 to 81.08%) which encode by the *aacA-D* gene (62.16%) is more prevalent amongst the human-based biotypes. It is because of this gene is usually more diffused in staphylococci of human origin [42]. Johler et al. (2011) [42] reported that prevalence of *ermC*, *tetK* and *tetM* antibiotic resistance genes among the *S. aureus* strains isolated from cases of food poisoning, milk and pork were 25, 4.87 and 0%, 50, 0 and 12.82 and 0%, 12.19, and 53.84%, respectively. Podkowik et al. (2012) [43] reported that the prevalence of tetracycline resistance genes (*tetO*, *tetK* and *tetM*) and erythromycin resistance methylase gene (*ermA*, *ermB* and *ermC*) among the *S. aureus* strains isolated from ready to eat meat products were 44 and 60%, respectively.

## Conclusions

The present investigation is the first report of the biotyping and study the antimicrobial resistance properties of MRSA strains isolated from raw and cooked hospital food samples. In this study, a total prevalence of 7.62% of MRSA as well as high prevalence of human and poultry-based biotypes was seen in hospital food samples. Considerable prevalence of resistance against penicillin, ceftaroline, tetracycline, erythromycin, trimethoprim-sulfamethoxazole, doxycycline and gentamicin antibiotic agents and high distribution of *tetK*, *ermA*, *msrA* and *aacA-D* antibiotic resistance genes may pose a potential public health threat. Our findings exhibited that the pattern of antibiotic resistance and also distribution of antibiotic resistance genes were dependent on the biotype of MRSA strains. Human-based biotypes had a higher prevalence of resistance against human-based antibiotics and their corresponding resistance genes. Raw food samples with animal origin had the lower prevalence of human-based biotypes, while cooked food samples had a higher prevalence of human-based biotypes. Presence of multi-drug resistance and also simultaneous presence of several antibiotic resistance genes in some MRSA isolates must be considered as serious health hazard. Cooking food thoroughly is important, but preventing cross-contamination is the most effective ways to prevent occurrence of MRSA in hospital food. Further researches are required to understand higher epidemiological aspects of the MRSA strains in hospital food samples.

## Abbreviations

MRSA: Methicillin-resistant *Staphylococcus aureus*
PCR: Polymerase chain reaction
*S. aureus*: *Staphylococcus aureus*
SPSS: Statistical package for the social sciences
